# Right dorsolateral prefrontal cortex regulates default prosociality preference

**DOI:** 10.1093/cercor/bhac429

**Published:** 2022-11-17

**Authors:** Hiroki Tanaka, Qiulu Shou, Toko Kiyonari, Tetsuya Matsuda, Masamichi Sakagami, Haruto Takagishi

**Affiliations:** Brain Science Institute, Tamagawa University, 6-1-1 Tamagawagakuen, Machida, Tokyo, 194-8610, Japan; Graduate School of Brain Sciences, Tamagawa University, 6-1-1 Tamagawagakuen, Machida, Tokyo, 194-8610, Japan; School of Social Informatics, Aoyama Gakuin University, 5-10-1 Fuchinobe, Sagamihara, Kanagawa, 252-5258, Japan; Brain Science Institute, Tamagawa University, 6-1-1 Tamagawagakuen, Machida, Tokyo, 194-8610, Japan; Brain Science Institute, Tamagawa University, 6-1-1 Tamagawagakuen, Machida, Tokyo, 194-8610, Japan; Brain Science Institute, Tamagawa University, 6-1-1 Tamagawagakuen, Machida, Tokyo, 194-8610, Japan

**Keywords:** brain structure, dorsolateral prefrontal cortex, economic game, social value orientation, voxel-based morphometry

## Abstract

The dorsolateral prefrontal cortex has been shown to be associated with prosocial behavior. However, the direction of this relationship remains controversial. To resolve inconsistencies in the existing literature, we introduced the concept of default prosociality preference and hypothesized that this preference moderates the relationship between gray matter volume in the dorsolateral prefrontal cortex and prosocial behavior. This study analyzed the data of 168 participants obtained from voxel-based morphometry, 4 types of economic games, and 3 different measures of social value orientation that represent default prosociality preference. Here we show that, in individuals who were consistently classified as proself on the 3 social value orientation measures, gray matter volume in the right dorsolateral prefrontal cortex was positively associated with prosocial behavior. However, in individuals who were consistently classified as prosocial, the direction of this association was vice versa. These results indicate that the right dorsolateral prefrontal cortex regulates default prosociality preference.

## Introduction

Humans exhibit a high degree of prosociality compared with that exhibited by other animals (Boyd and Richerson 2009; Bowles and Gintis 2011; Nowak and Highfield 2011; Tomasello et al. 2012), and the psychological and neural mechanisms underlying human prosocial behavior have attracted considerable research interest. Findings regarding the role of the dorsolateral prefrontal cortex (DLPFC), a brain region involved in cognitive control, such as self-control and inhibitory control (Miller and Cohen 2001; Hare et al. 2009; Figner et al. 2010; Schmidt et al. 2018), in human prosocial behavior have been inconsistent. Several studies have shown that DLPFC-mediated cognitive control promotes prosocial behavior (Knoch et al. 2006; Steinbeis et al. 2012; Ruff et al. 2013) and that the suppression of DLPFC function results in reduced prosocial behavior (Knoch et al. 2006; Ruff et al. 2013). Furthermore, individuals with greater DLPFC thickness engage in more prosocial behavior (Steinbeis et al. 2012). Contrarily, DLPFC-mediated cognitive control has also been reported to play a role in inhibiting prosocial behavior. Individuals with greater DLPFC volume or thickness and greater capacity for strategic thinking are more likely to show selfish behavior (Fermin et al. 2016; Yamagishi et al. 2016). Hence, the role of DLPFC-mediated cognitive control in prosociality remains unclear.

Yamagishi et al. (2017) found that the relationship between response time (RT) and overall prosocial behavior was reversed according to the participants’ social value orientation (SVO), which represents a social preference for choice of reward distribution between self and others (Van Lange et al. 1997). This preference can be expressed on a 1-dimensional scale between favoring more self-maximizing distribution and favoring more equitable distribution, and can be classified as proself and prosocial according to the degree observed. For those classified as proself, longer RT indicated greater prosocial behavior. However, for those classified as prosocial, shorter RT indicated greater prosocial behavior (Yamagishi et al. 2017). According to a previous study on the association between RT and the social preference in value-based decision-making, length of RT reflects a conflict between strength of preference and actual choice (Krajbich et al. 2015). Therefore, results of Yamagishi et al. (2017) imply that the conflict in decision-making occurs when people need to make a decision that deviates from their social preference regardless of their SVO. Specifically, the conflict between individuals’ social preference and prosocial behavior occurs when their SVO is proself, whereas prosocials have a conflict when they refrain from prosocial behavior.

**Fig. 1:**
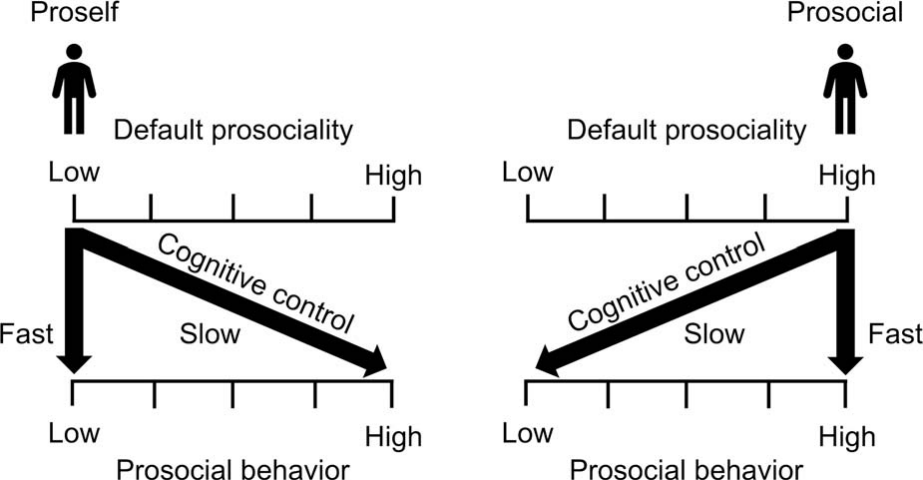
Schematic representation of the relationship between the default preference for prosociality and prosocial behaviors. In proselfs, the default preference for prosociality is low; therefore, selfish behavior, owing to consistency, can be performed quickly. However, when they engage in prosocial behavior, it takes time because they need to cognitively control their default preferences that are not consistent with prosocial behavior. In prosocials, the default preference for prosociality is high, and they are able to quickly perform prosocial behavior consistent with it. However, when they do not engage in prosocial behavior, it takes time because they need to cognitively control their default preferences that are not consistent with selfish behavior.

Given that SVO indicates the degree of readiness for prosocial behavior or default prosociality preference, we proposed the following model. If default prosociality is high, people are classified as prosocial in SVO and they can immediately engage in prosocial behaviors because this decision is consistent with default prosociality. Conversely, if it is low, people are classified as proself and would require more time to engage in prosocial behaviors because the decision is inconsistent with default prosociality, and this preference needs to be cognitively controlled. Therefore, the direction of the association between RT and prosocial behavior would be reversed depending on default prosociality. A schematic representation of this relationship is shown in Fig. 1.

With regard to the direction of the association between the role of DLPFC-mediated cognitive control and prosocial behavior, the results predicted by our model differ from those in studies that found DLPFC to be positively (Knoch et al. 2006; Steinbeis et al. 2012; Ruff et al. 2013) or negatively associated with prosocial behavior (Fermin et al. 2016; Yamagishi et al. 2016). Rather, our model predicts that DLPFC-mediated cognitive control may influence the default prosociality preference, regardless of whether it is high (i.e. prosocial) or low (i.e. proself), and lead to behavior that deviates from the preference. Specifically, in proselfs, DLPFC allows prosocial behavior by regulating the low default prosociality. In contrast, DLPFC allows prosocials to refrain from prosocial behavior by regulating the high default prosociality. To test these predictions, this study examined whether the effect of DLPFC volumes on prosocial behavior is moderated by SVO. According to previous studies, the volume of DLPFC positively reflected cognitive control, such as self-control (Schmidt et al. 2018; Xu et al. 2021) and strategic behavior (Fermin et al. 2016). We hypothesized that the relationship between prosocial behavior and the DLPFC volume would be reversed by SVO. We predicted a higher degree of prosocial behavior in proself individuals with a larger DLPFC volume and in prosocial individuals with a smaller DLPFC volume.

## Materials and methods

### Participants

We performed a secondary analysis of data from databases built during a previous research project, “Neuropsychological and Social Institutional Foundations for Prosocial Behavior” (http://www.human-sociality.net/english/), conducted between 2012 and 2018. Approximately 400 adults participated in the project, which involved a series of experiments in which they played economic games measuring prosocial behavior and completed various psychological scales and cognitive tasks. Furthermore, magnetic resonance imaging (MRI) and genetic polymorphism data were collected. To avoid per-experiment burden on participants and the carryover effect of the economic games, the tasks were conducted on different days. Supplementary Fig. 1 summarizes the dates of the assignments (see online supplementary material for a color version of the figure). On the basis of the hypothesis generated from the results of a previous study (Yamagishi et al. 2017), the present study reanalyzed prosocial behavior, including MRI data. The data collected in this project have been reported in many papers (Supplementary Table 1). However, this is the first study to investigate whether DLPFC volume and prosocial behavior are inversely related according to SVO of the participants. All experimental protocols were approved by the ethics committee of Tamagawa University (Tokyo, Japan; approval no. TRE18-036). Each participant signed an informed consent form before the experiment.

### Social value orientation

Data from 3 different SVO scales were used: the 9-item Triple-Dominance Scale (Van Lange et al. 1997), 24-item Ring Scale (Liebrand 1984), and 15-item Slider Scale (Murphy et al. 2011). Using a computer, participants responded to preferences regarding the distribution of rewards between themselves and others. SVO classification was based on the criteria of the respective scale. The Triple-Dominance Scale classified participants who chose 6 or more equal distribution options out of 9 options as prosocial, and those who chose 6 or more options with the greatest self-interest or the greatest difference between self-interest and others’ interests as proself. For the 24-item Ring Scale and the 15-item Slider Scale, the angle was calculated from the degree of weighting for the value of the interests of self and others, and participants who showed >22.5 degrees (22.45 degrees for Slider Scale) were classified as prosocial, whereas those who showed <22.5 degrees (22.45 degrees for Slider Scale) were classified as proself. In a previous study (Yamagishi et al. 2017), the association between RT and prosocial behavior was more pronounced among those who were consistently classified as prosocial or proself on each SVO scale. Based on this, the final analysis included this consistent classification into prosocial/proself individuals.

### Dictator game

The dictator game (DG) involves pairs containing an allocator and a recipient. The allocator decides how to divide the money received from the experimenter between themselves and the recipient. Participants first played one DG using paper and pencil. All participants played the role of the allocator and decided how they would divide JPY 1,000 (USD 8.65) between themselves and an anonymous partner. After the game using paper and pencil was over, participants then used their computers for the DG. Each participant played the game in 6 trials, changing partners each time. The endowments for each trial were as follows: JPY 300, JPY 400, JPY 600, JPY 700, JPY 1,200, and JPY 1,300. All endowments were presented at random. After all the trials were finished, participants’ roles and those of the opponents were randomly determined, and the reward was fixed according to participants’ actual game behavior. In the DG, the mean proportion of 7 allocations (1 using paper and pencil and 6 using the computer) was used as a measure of prosocial behavior.

### Prisoner’s dilemma game I

The prisoner’s dilemma game (PDG) is an economic game played in pairs. In the first PDG (PDG-I), participants had to decide whether they would give the money they received from the experimenter to an anonymous partner. The money provided was doubled and given to the other player. Participants participated in repeated PDGs with different partners in the following 3 conditions: (i) simultaneous condition: both players decide at the same time; (ii) sequential-first condition: the participant decides first, and the other player observes the decision of the participant and makes the decision whether he or she will give money to their partner; and (iii) sequential-second condition: the other player makes a decision first, and the participant observes the decision of the other player and then makes a decision. The third condition used the strategy method, in which the participants answered the decision in 2 situations: (i) the other player decided to provide money; or (ii) the other player declined to provide money. Three different endowments were received from the experimenter for each of the 3 conditions (JPY 300, JPY 800, and JPY 1,500). In PDG-I, the provision rate in all trials was used as an indicator of prosocial behavior.

### Prisoner’s dilemma game II

In the second PDG (PDG-II), the experimenter gave the participants JPY 1,000 each. Using increments of JPY 100, the participants had to decide how much of this they would give to another player. The chosen amount was then doubled and given to the other player. The other player also decided at the same time. Both players earned JPY 2,000 if they gave the full amount and JPY 1,000 if they did not give any at all. The game was completed in 1 session and participants were rewarded based on their actual behavior. In the PDG-II, the amount provided by the participants was used as an indicator of prosocial behavior.

### Public goods game

The public goods game (PGG) is an economic game played by multiple players simultaneously. Each participant received JPY 1,000 from the experimenter and decided how much of it to offer to the group. All contributions were summed, doubled, and distributed equally. The experiment was completed in 1 session. The PGGs were played in groups of at least 4 people. The amount of money offered by the participants was used as an indicator of prosocial behavior.

### Trust game

The trust game (TG) was played by 2 parties, the trustor and trustee. The trustor decided how much of the JPY 1,000 they received from the experimenter would be transferred to the other player in increments of JPY 100. Then, the trustee received the tripled transfer amount. The trustee distributed the money between themselves and the trustor. All participants in the experiment were first given the role of the trustor. Subsequently, the paired partners were changed, and they were given the role of the trustee. Trustee decisions were made using the strategy method regarding how to distribute the money for all possible trustor behaviors. Based on the previous research (Yamagishi et al. 2017), the mean allocation rate by the trustee was used as an indicator of prosocial behavior.

### Overall prosocial behavior

Values of distribution in the DG, cooperation in PDG-I and PDG-II, provision in the PGG, and trustee distribution in the TG were standardized and averaged. The present study examined the associations between them with brain imaging as a measure of overall prosocial behavior. The correlations of behavior in each economic game are shown in Supplementary Table 2.

### MRI data collection

MRI data were collected using a 3T Siemens Trio A Tim MRI scanner. High-resolution anatomical images were acquired using a T1-weighted 3D magnetization prepared rapid acquisition gradient echo sequence (repetition time, 2,000 ms; echo time, 1.98 ms; field of view, 256 × 256 mm; number of slices, 192; voxel size, 1 × 1 × 1 mm; average, 3 times).

### MRI data analysis

Computational anatomy toolbox (CAT12, http://dbm.neuro.uni-jena.de/cat/) and statistical parametric mapping software (SPM12, http://www.fil.ion.ucl.ac.uk/spm) were used to process and analyze the T1-weighted images. Images were corrected for bias, marked for tissue or fluid type (gray matter [GM], white matter, and cerebrospinal fluid), and registered using linear (12-parameter affine) and nonlinear (warp) transformations within the default preprocessing pipeline of CAT12.

In the first step, modulated normalized images were generated and smoothed by the standard SPM12 smoothing pipeline with a smoothing kernel of 8 × 8 × 8-mm full width at half-maximum, and CAT12 was used to estimate the overall volume of GM, white matter, and cerebrospinal fluid. Using the preprocessed data, whole-brain analyses were conducted to examine regions showing interaction effects between SVO and prosocial behavior. In particular, we examined brain regions that were positively correlated with prosocial behavior in proself individuals, and vice versa in prosocial individuals. We analyzed the interaction terms of prosocial behavior, SVO (0 = proself, 1 = prosocial) and overall prosocial behavior and SVO as explanatory variables, with age, sex, and total GM volume as covariates. The results of the whole-brain analysis were examined for brain regions that were significant at the 0.05 threshold in the family-wise error (FWE) correction. The coordinates indicated by SPM12 were changed to Talairach coordinates by GingerALE (Eickhoff et al. 2009), and the Talairach Client Version 2.4.3 (http://www.talairach.org/client.html) was used to identify the brain region.

### Statistical analysis

All analyses reported in this study were performed using SAS 9.4. All analyses included participants who consistently classified as prosocials/proselfs in each SVO scale (triple-dominance, ring, and slider), and for whom there were data available from DG, PDG-I, PDG-II, PGG, TG, and T1-weighted imaging. Finally, data of 168 participants (men = 100, women = 68, age_mean_ = 40.5 years, age_SD_ = 10.2 years, age_range_ = 20–59 years) were used. Of these, 79 participants were prosocials and 89 were proselfs.

## Results

Overall prosocial behavior tended to increase with age (*r*_(166)_ = 0.25, *P* = 0.001), but it did not differ by sex (men: *M* = 0.025, *SD* = 1.08; women: *M* = −0.037, *SD* = 0.87, *t*_(166)_ = 0.4, *P* = 0.691). Further, prosocial behavior tended to be higher among prosocial individuals (prosocials: *M* = 0.78, *SD* = 0.72, proselfs: *M* = −0.69, *SD* = 0.65, *t*_(166)_ = 13.9, *P* < 0.0001, *d* = 2.1).

**Fig. 2:**
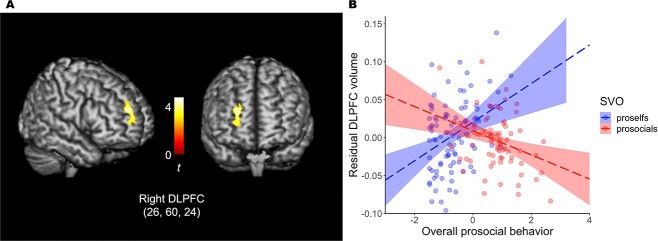
Results of voxel-based morphometry analysis showing the interaction effect of social value orientation and prosocial behavior. A) Whole-brain analysis reveals that the volume of the right dorsolateral prefrontal cortex (DLPFC) (26, 60, 24) shows an interaction effect between social value orientation and prosocial behavior (*P*_FWE_ < 0.05). B) The larger is the volume of the right DLPFC in proselfs, the higher is the level of prosocial behavior. Conversely, the smaller is the volume of the right DLPFC in prosocials, the higher is the level of prosocial behavior. The volume of the right DLPFC is used in the analysis after controlling for age, sex, and total gray matter volume. The blue and red areas represent 95% confidence intervals. SVO = social value orientation.

The voxel-based morphometry analysis showed the involvement of the right DLPFC volume, even after multiple comparison correction (peak coordinates: *x* = 26, *y* = 60, *z* = 24, *t* = 4.77, *z* = 4.61, peak *P*_FWE_ = 0.049, cluster size = 776; Fig. 2A). The GM volume within a 10-mm radius of the peak coordinates was extracted and analyzed for each SVO, controlling for age, sex, and total GM volume. In proself individuals, we observed that the larger the GM volume of the right DLPFC, the more prosocial the behavior (*r*(87) = 0.36, *P* < 0.001; Fig. 2B). Conversely, in prosocial individuals, we observed that the lesser the GM volume of the right DLPFC, the more prosocial the behavior (*r*(77) = −0.33, *P* = 0.003). Similar interaction effects were found for each economic game (DG: β = −0.24, *P* = 0.005, Supplementary Fig. 2; PDG-I: β = −0.21, *P* = 0.010, Supplementary Fig. 3; PDG-II: β = −0.23, *P* = 0.043, Supplementary Fig. 4; PGG: β = −0.23, *P* = 0.008, Supplementary Fig. 5; TG: β = −0.30, *P* < 0.001, Supplementary Fig. 6, see online supplementary material for a color version of these figures). Table 1 shows the results of the whole-brain analysis with uncorrected *P*-values < 0.001. Although the medial prefrontal cortex (MPFC), left DLPFC, and caudate showed similar patterns to that of the right DLPFC, these patterns did not survive after correction for multiple comparisons. Furthermore, no brain regions were found to be negatively associated with prosocial behavior in proself individuals or positively associated with prosocial behavior in prosocial individuals.

**Table 1:** Whole-brain analysis of the interaction effect of prosocial behavior and social value orientation (voxels survived at *P* < 0.001 uncorrected with an extent of > 78 voxels).

Anatomical location	BA	L/R	Peak MNI coordinates			Peak	Peak	Peak	Cluster	Cluster
						*t*-value	*z*-value	*P*-value	size	*P*-value
			*x*	*y*	*z*			(FWE corrected)		(FWE corrected)
Superior frontal gyrus	9	R	26	60	24	4.77	4.61	0.049	776	0.029
Middle frontal gyrus	10	R	28	63	9	3.94	3.84	0.580		
Superior frontal gyrus	8	R	20	56	32	3.70	3.62	0.835		
Caudate		L	−3	12	3	4.11	4.00	0.398	212	0.524
Medial frontal gyrus	10	L	0	64	−10	4.04	3.94	0.467	287	0.355
Medial frontal gyrus	10	L	0	54	−16	3.64	3.57	0.879		
Superior frontal gyrus	10	L	−22	62	−2	3.89	3.79	0.644	157	0.680

Abbreviations: MNI, Montreal Neurological Institute; FWE, family-wise error; L, left; R, right; BA, Brodmann area.

## Discussion

This study revealed that the association between right DLPFC volume and prosocial behavior varied in its direction depending on the participants’ SVO. In proself individuals, the larger the volume of the right DLPFC, the more prosocial the behavior. Conversely, in prosocial individuals, the smaller the volume of the right DLPFC, the more prosocial the behavior. Based on previous evidence implicating the DLPFC as a region involved in cognitive control (Miller and Cohen 2001; Hare et al. 2009; Figner et al. 2010; Schmidt et al. 2018), the present results support our hypothesis that the right DLPFC plays a role in controlling default preferences for prosociality. Specifically, proself individuals have a low default prosociality preference and need to regulate it when performing prosocial behavior using right DLPFC. Meanwhile, prosocial individuals have a high default prosociality preference and need to regulate it when inhibiting prosocial behavior using right DLPFC. Although one previous study (Abe and Greene 2014) has already shown that DLPFC activity expresses the difference between one’s values and actual behavior, our study provides more detailed findings regarding the role of the DLPFC in social decision-making.

In the current study, we focused on the association between DLPFC and prosocial behavior in prosocial and proself individuals. It remains unclear whether the structure of brain regions, including the DLPFC, reflects the level of function. However, the structure of the DLPFC has been commonly used to assess cognitive control function (Weinstein et al. 2012) and examine its association with behavior in economic games (Steinbeis et al. 2012; Fermin et al. 2016; Yamagishi et al. 2016). One possible explanation for larger DLPFC volumes is experience-dependent expansion as a result of frequent activity (Maguire et al. 2006). In previous studies, the volume of GM of DLPFC was positively associated with self-control and strategic behavior (Fermin et al. 2016; Schmidt et al. 2018; Xu et al. 2021). Therefore, it is reasonable to assume that DLPFC volume is associated with that brain region’s activity and level of function. Our study indicated that structural aspects influenced the differences in the DLPFC’s association with prosocial behavior in prosocial and proself individuals. To directly demonstrate our hypothesis, future studies should examine the functional role of the DLPFC.

Our results may explain the inconsistencies in the existing literature concerning the role of the DLPFC in prosocial behavior (Knoch et al. 2006; Ruff et al. 2013; Fermin et al. 2016; Yamagishi et al. 2016). For instance, if most of a study’s participants were classified as proself, the study results would show that the right DLPFC would play a role in facilitating prosocial behavior since the DLPFC suppresses default prosociality (Knoch et al. 2006; Steinbeis et al. 2012; Ruff et al. 2013). Furthermore, prosocial behavior decreases when DLPFC functions are externally inhibited by noninvasive stimulations such as low-frequency repetitive transcranial magnetic stimulation and transcranial direct current stimulation (Knoch et al. 2006; Ruff et al. 2013). Conversely, if most study participants were prosocial, the right DLPFC would play a role in inhibiting prosocial behavior (Fermin et al. 2016; Yamagishi et al. 2016). Therefore, prosocial behavior would be promoted when right DLPFC function is inhibited. This phenomenon of reversal of results depending on participant SVO is worthy of consideration. Future experiments should be carefully conducted focusing on participants’ default prosociality preferences.

From the perspective of controlling default preferences, the discrepancy in the results between adults and children can be well explained (Steinbeis et al. 2012; Fermin et al. 2016; Yamagishi et al. 2016). One study showed that children who engaged in prosocial behavior had increased DLPFC activity and thicker cortices (Steinbeis et al. 2012). Studies on adults showed the opposite. Those with decreased DLPFC activity and thinner cortices engaged in more prosocial behavior (Fermin et al. 2016; Yamagishi et al. 2016). Many studies have shown that children make selfish choices in resource allocation situations more often than do adults (Fehr et al. 2008; Takagishi et al. 2010, 2014, 2015). This suggests that default prosociality is lower in children, and it is necessary to control that default preference to achieve prosocial behavior. Presumably, during developmental interactions with the social environment, a high level of default prosociality in some individuals may have resulted in the patterns seen in the adult studies. No studies have yet examined how the relationship between RT and prosocial behavior changes over the course of development, and it is not clear why some adults have higher default prosociality than that of others. We believe it is necessary to investigate individual differences, including social environmental factors, in default prosociality in the future.

In addition, recent studies have frequently shown that brain functions in the right hemisphere regions, such as the temporoparietal junction (TPJ), are important for prosocial behavior (Morishima et al. 2012; Kameda et al. 2016). The parietal lobes, including the right TPJ, together with the right DLPFC, belong to the frontoparietal network, which is a key component of cognitive control and has been shown to have strong functional connections (Zanto and Gazzaley 2013). These findings indicate that the right-hemispheric-dominant brain neural circuits may support prosocial behavior. Recent studies have shown that proactive cognitive control is associated with the left DLPFC and reactive cognitive control is associated with the right DLPFC. These findings indicate that the regions responsible for cognitive control differ depending on the type of behavior (Pulopulos et al. 2022). Based on these results, it is likely that the prosocial behavior exhibited by humans involves reactive cognitive control; however, no studies directly demonstrate this. Hence, future research is needed.

This study has some limitations. It should be noted that the conducted analysis did not use a neuroscientific state but a trait as the independent variable. That is, the present study found that the level of prosocial behavior among individuals with greater right DLPFC volume deviated from their default preferences, and did not investigate whether proselfs/prosocials switch to higher/lower levels of their prosocial behavior depending on DLPFC activity within individuals. However, Hackel et al. (2020) found that right DLPFC activity and DLPFC-ventromedial prefrontal cortex (VMPFC) functional connectivity were observed when individuals made a social decision that deviated from their default prosocial preferences. Given that the VMPFC is thought to be involved in the representation of subjective value computation (Levy and Glimcher 2012; Bartra et al. 2013), Hackel et al. (2020) discussed that the DLPFC may switch between cooperative and non-cooperative behavior by regulating subjective value computed in the VMPFC during social decision-making. How DLPFC activity patterns relate to DLPFC volume, whether DLPFC volume is associated with constant suppression of default preferences or with flexible switching of cooperative and non-cooperative behavior, and under what social conditions prosocial/proself preferences are regulated by DLPFC activity need to be examined in future studies.

In conclusion, our study examined the role of DLPFC in prosocial behavior by assessing the adjustment effect of SVO. To our knowledge, this is the first study to demonstrate the reversed effect of the right DLPFC on prosocial behavior depending on SVO. Therefore, we posit that SVO can be used to explain the inconsistent findings in previous studies, and this current study could provide new directions for future studies. Moreover, since our study identified prosocial behavior using various economic games, its conclusions could be applied to prosocial behaviors including generosity, reciprocity, and altruism.

## Supplementary Material

Supplementary_materials_20221006_bhac429

Dataset_bhac429

## Data Availability

All relevant data are included within this article and its supplementary data.

## Notes

We thank Toshio Yamagishi, Ryota Kanai, Kuniyuki Nishina, Alan Fermin, Yoshie Matsumoto, and Yang Li for their help in conducting the study.
