# Supplementary material for: Right dorsolateral prefrontal cortex regulates default prosociality preference
Source: Cereb Cortex. 2022 Nov 17;33(9):5420–5. doi: 10.1093/cercor/bhac429 (PMC10152081; doi:10.1093/cercor/bhac429)
Supplement: Supplementary_materials_20221006_bhac429 [file supplementary_materials_20221006_bhac429.docx]

**Supplementary materials**

Right dorsolateral prefrontal cortex regulates default preference for prosociality

Hiroki Tanaka, Qiulu Shou, Toko Kiyonari, Tetsuya Matsuda, Masamichi Sakagami, Haruto Takagishi


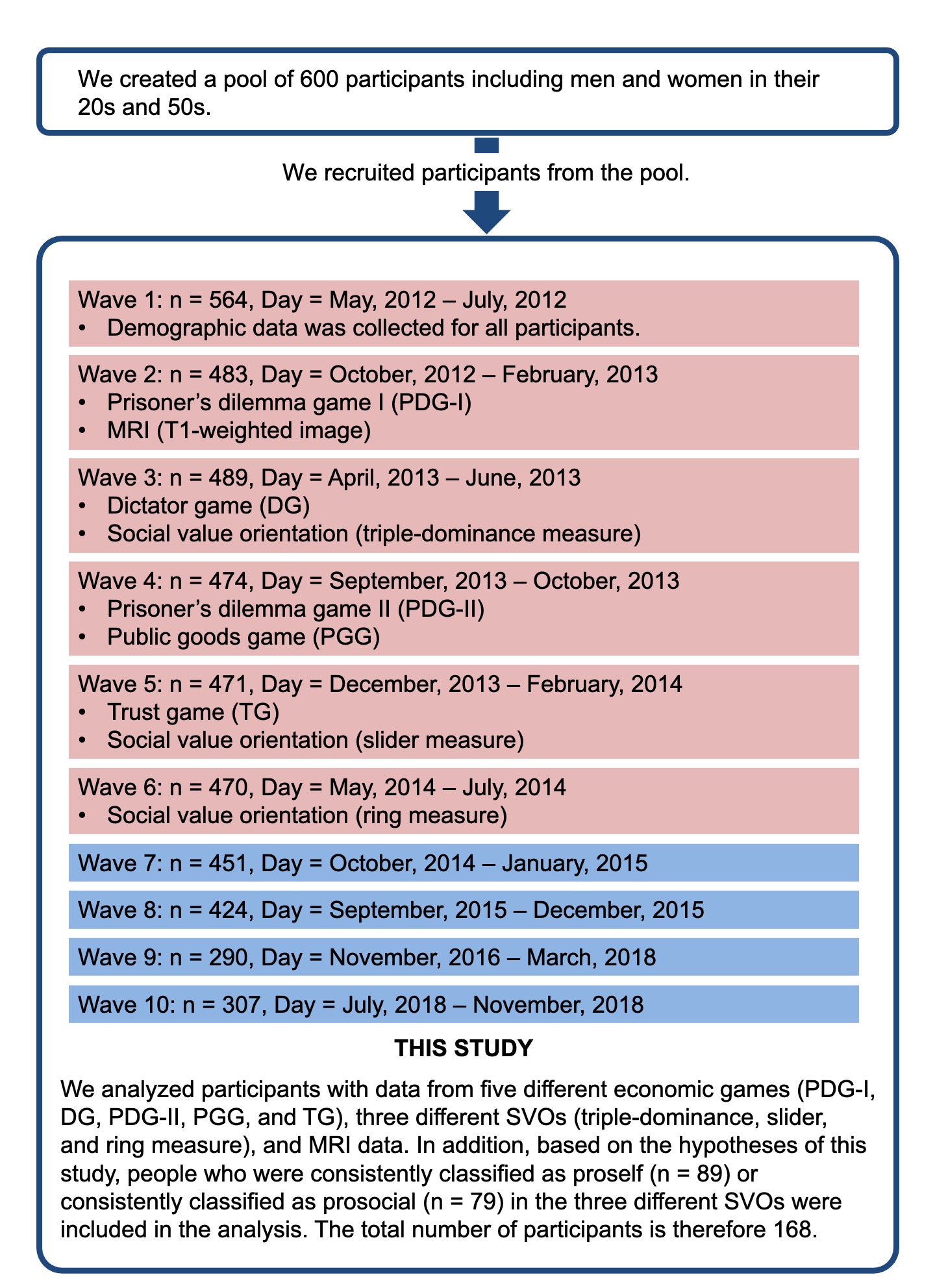


Figure S1. Outline of the research project

SVO = social value orientation


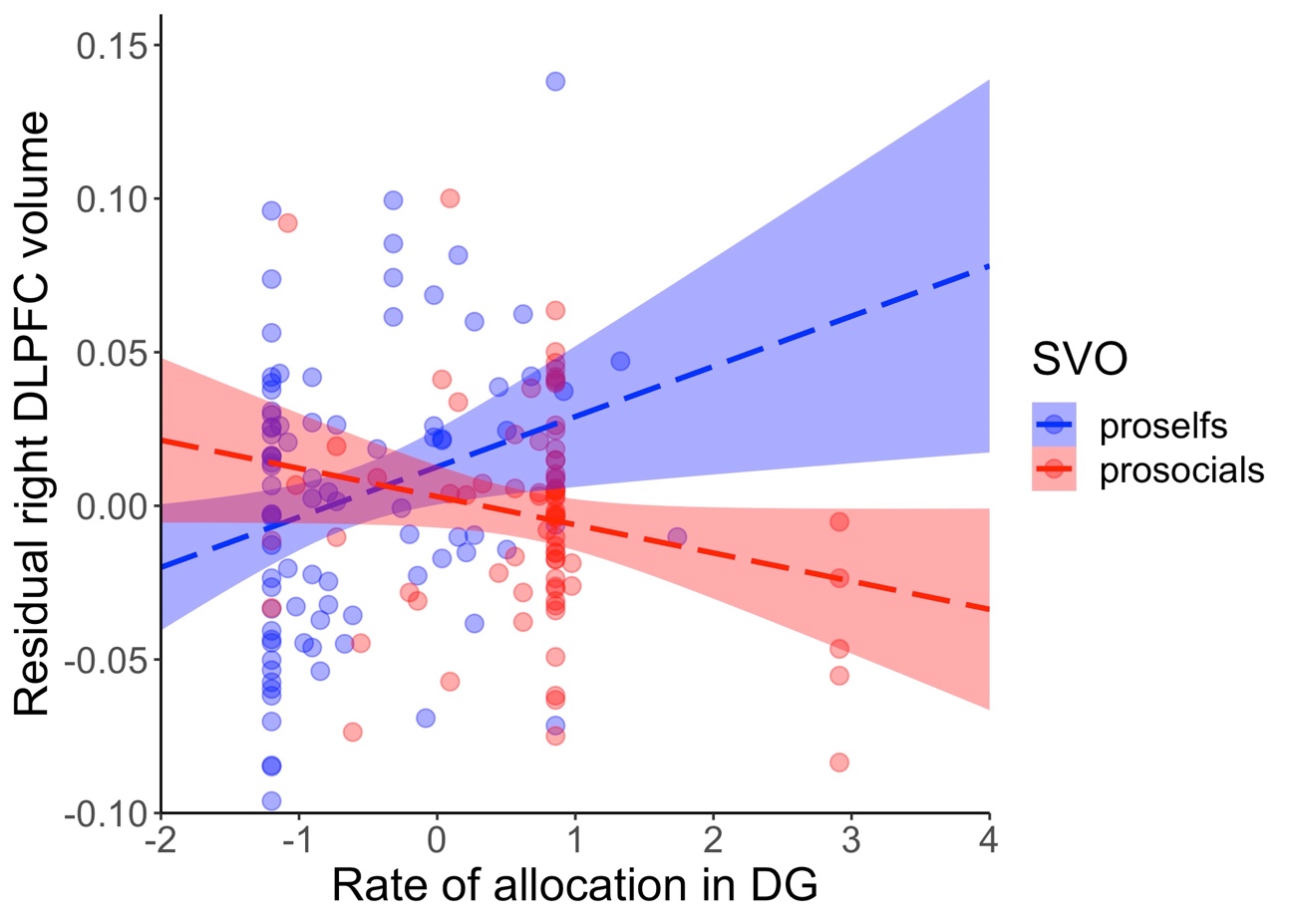


Figure S2. Relationship between the rate of allocation in the DG and residual right DLPFC volume for each SVO. The volume of the DLPFC was controlled for age, sex, and total grey matter volume.

DG = dictator game, DLPFC = dorsolateral prefrontal cortex, SVO = social value orientation


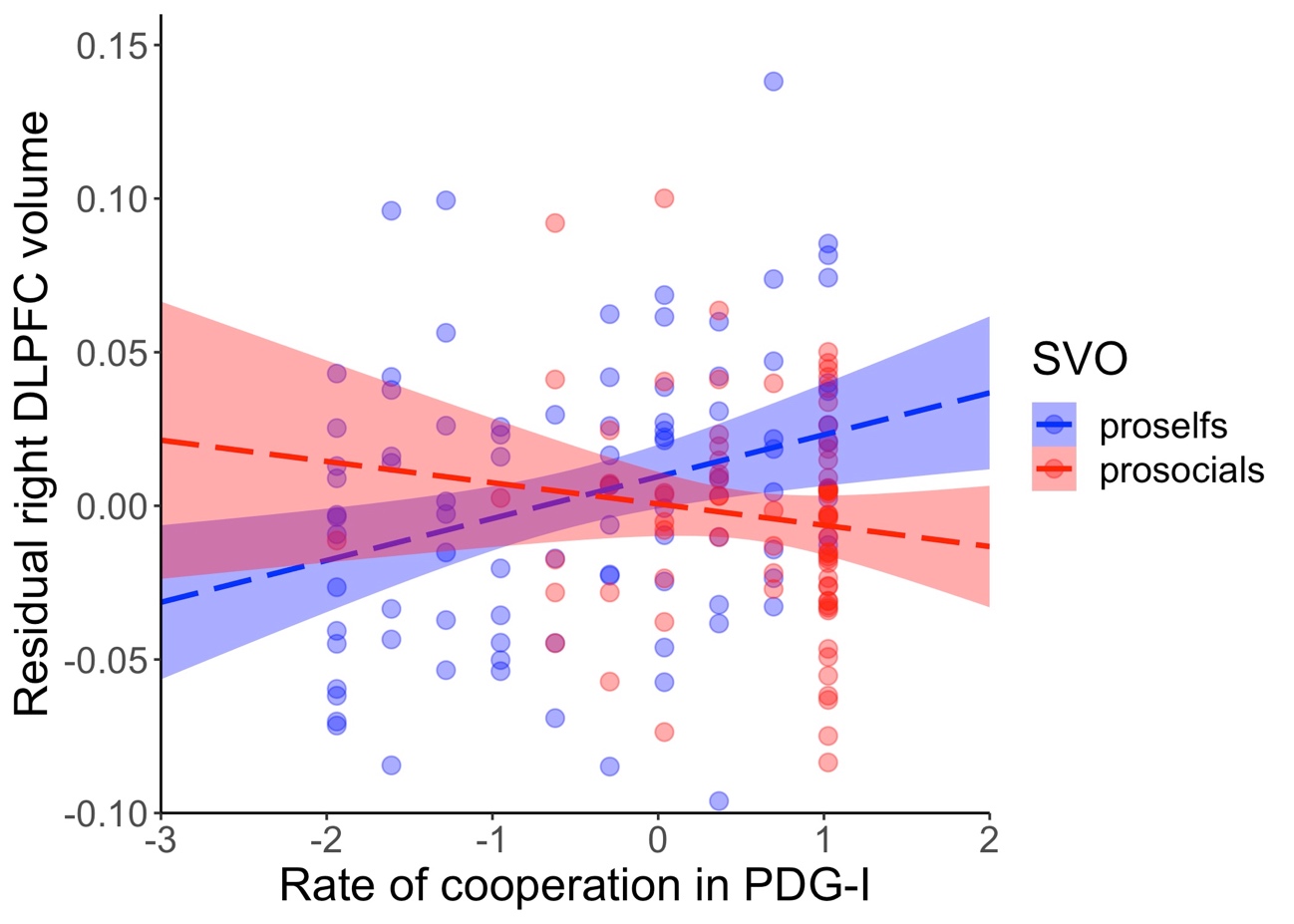


Figure S3. Relationship between the rate of cooperation in the PDG-I and residual right DLPFC volume for each SVO. The volume of the DLPFC was controlled for age, sex, and total grey matter volume.

PDG = prisoner’s dilemma game, DLPFC = dorsolateral prefrontal cortex, SVO = social value orientation


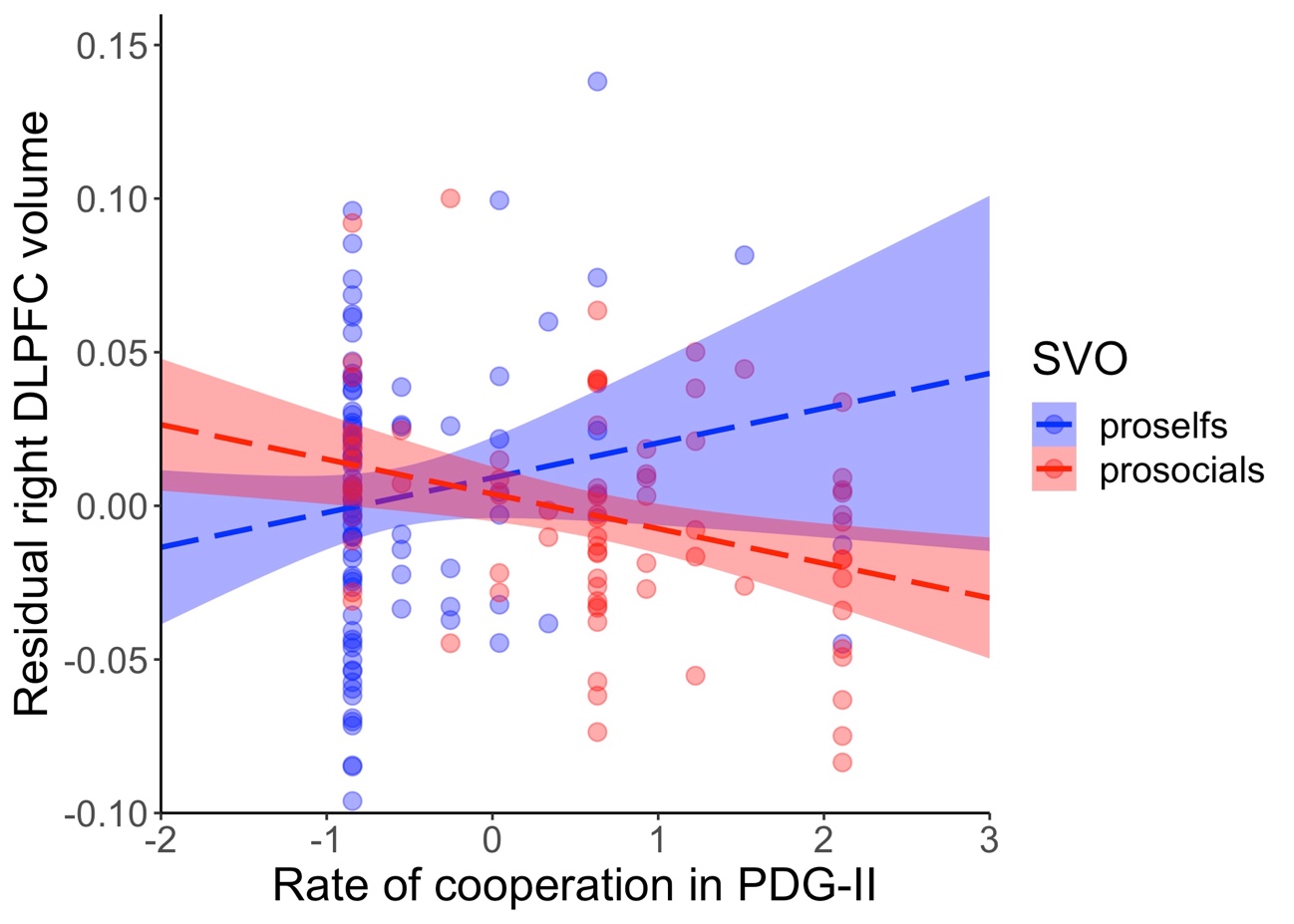


Figure S4. Relationship between the rate of cooperation in the PDG-II and residual right DLPFC volume for each SVO. The volume of the DLPFC was controlled for age, sex, and total grey matter volume.

PDG = prisoner’s dilemma game, DLPFC = dorsolateral prefrontal cortex, SVO = social value orientation


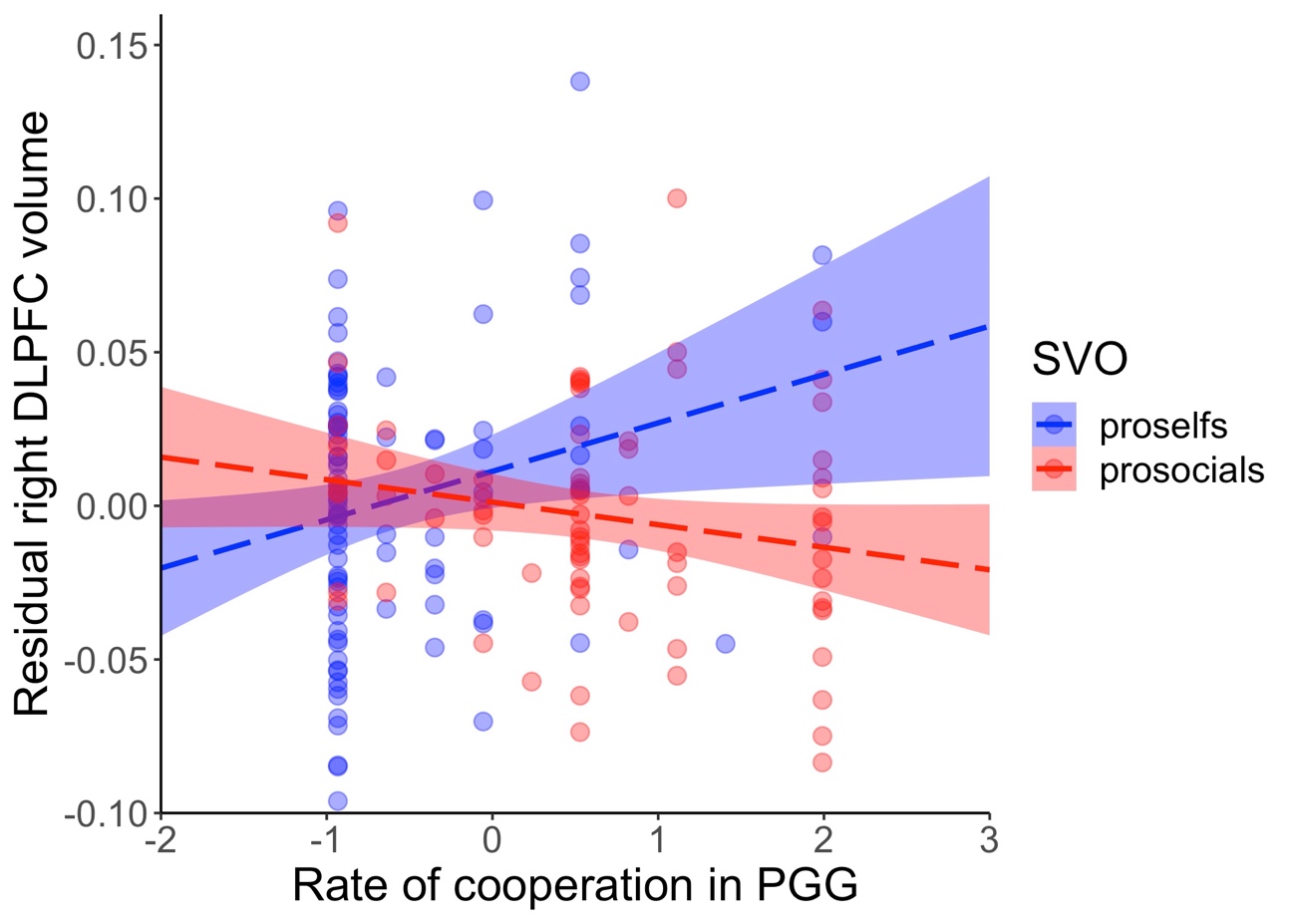


Figure S5. Relationship between the rate of cooperation in the PGG and residual right DLPFC volume for each SVO. The volume of the DLPFC was controlled for age, sex, and total grey matter volume.

PGG = public goods game, DLPFC = dorsolateral prefrontal cortex, SVO = social value orientation


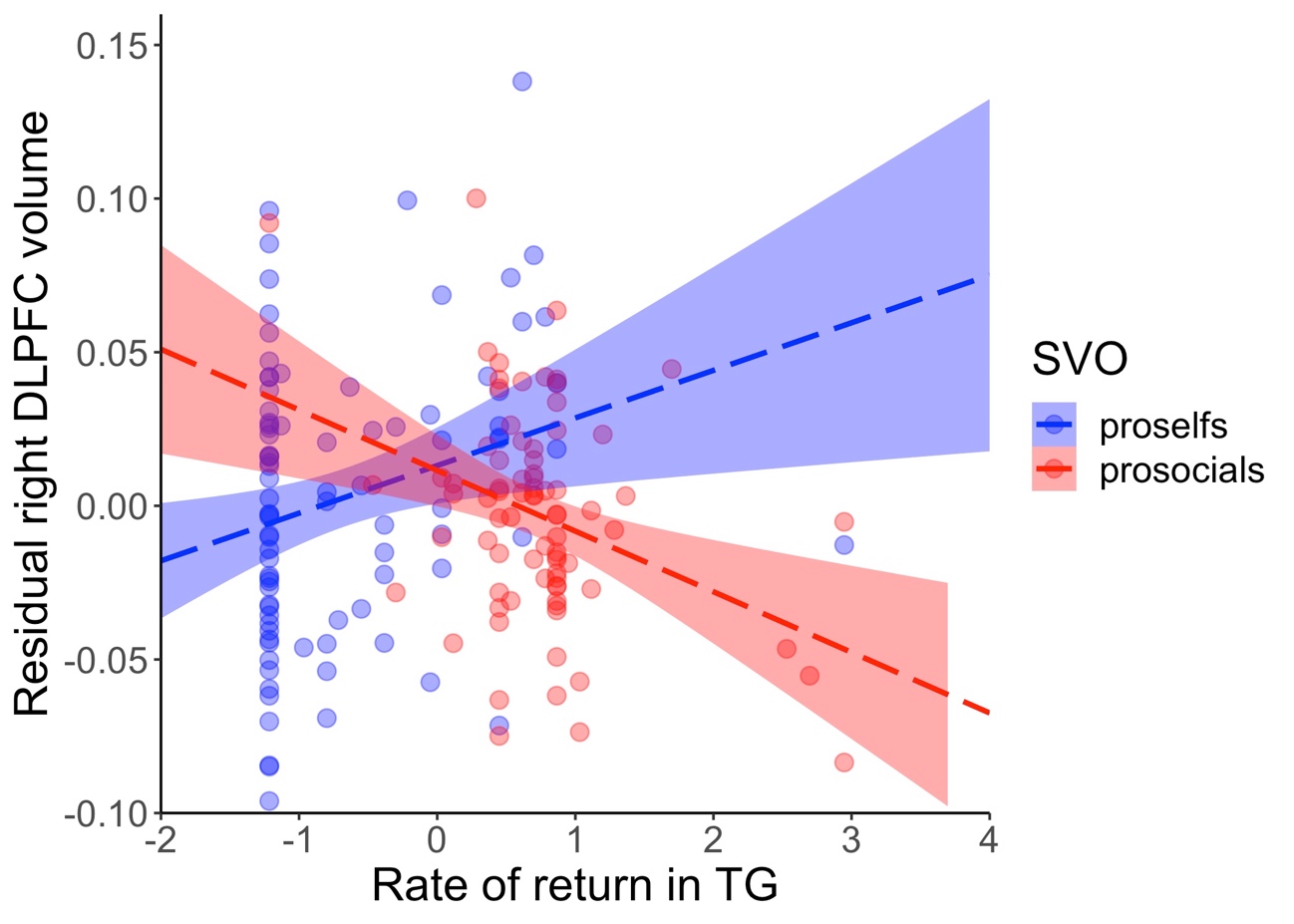


Figure S6. Relationship between the rate of return in the TG and residual right DLPFC volume for each SVO. The volume of the DLPFC was controlled for age, sex, and total grey matter volume.

TG = trust game, DLPFC = dorsolateral prefrontal cortex, SVO = social value orientation

| Table S1. List of papers published in this research project | |
| --- | --- |
| No. | Bibliographic information of the article |
| 1. | Shou Q, Yamada J, Nishina K, Matsunaga M, Matsuda T, Takagishi H. (2022). Association between salivary oxytocin levels and the amygdala and hippocampal volumes. *Brain Struct Funct*, 227, 2503-2511. |
| 2. | Shou Q, Yamada J, Nishina K, Matsunaga M, Kiyonari K, Takagishi H. (2022). Is oxytocin a trust hormone? Salivary oxytocin is associated with caution but not with general trust. *PLoS One*, 17(5): e0267988. |
| 3. | Nishina K, Shou Q, Takahashi H, Sakagami M, Inoue-Murayama M, Takagishi H. (2022). Association Between Polymorphism (5-HTTLPR) of the Serotonin Transporter Gene and Behavioral Response to Unfair Distribution. *Front Behav Neurosci*, 16: 762092. |
| 4. | Yamada J, Nakawake Y, Shou Q, Nishina K, Matsunaga M, Takagishi H. (2021). Salivary Oxytocin Is Negatively Associated With Religious Faith in Japanese Non-Abrahamic People. *Front Psychol*, 12: 705781. |
| 5. | Ishihara T, Miyazaki A, Tanaka H, Fujii T, Takahashi M, Nishina K, Kanari K, Takagishi H, Matsuda T. (2021). Childhood exercise predicts response inhibition in later life via changes in brain connectivity and structure. *NeuroImage*, 237, 118196. |
| 6. | Nishina K, Takagishi H, Takahashi H, Sakagami M, Inoue-Murayama M. (2019). Association of polymorphism of arginine-vasopressin receptor 1A (AVPR1a) gene with trust and reciprocity. *Front Hum Neurosci*, 13, 230. |
| 7. | Nishina K, Takagishi H, Fermin ASR, Inoue-Murayama M, Takahashi H, Sakagami M, Yamagishi T. (2018). Association of the oxytocin receptor gene with attitudinal trust: role of amygdala volume. *Soc Cogn Affec Neurosci*, 13, 1091-1097. |
| 8. | Yamagishi T, Li Y, Fermin AS, Kanai R, Takagishi H, Matsumoto Y, Kiyonari T, Sakagami M. (2017). Behavioural differences and neural substrates of altruistic and spiteful punishment. *Sci Rep*, 7, 14654. |
| 9. | Yamagishi T, Matsumoto Y, Kiyonari T, Takagishi H, Li Y, Kanai R, Sakagami M. (2017). Response time in economic games reflects different types of decision conflict for prosocial and proself individuals. *Proc Natl Acad Sci U S A*, 114, 6394-6399. |
| 10. | Yamagishi T, Takagishi H, Fermin ASR, Kanai R, Li Y, Matsumoto Y. (2016). Cortical thickness of the dorsolateral prefrontal cortex predicts strategic choices in economic games. *Proc Natl Acad Sci U S A*, 113, 5582-5587. |
| 11. | Matsumoto Y, Yamagishi T, Li Y, Kiyonari T. (2016). Prosocial behavior increases with age across five economic games. *PLoS One*, 11, e0158671. |
| 12. | Yamagishi T, Li Y, Matsumoto Y, Kiyonari T. (2016). Moral bargain hunters purchase moral righteousness when it is cheap: within-individual effect of stake size in economic games. *Sci Rep*, 6, 27824. |
| 13. | Nishina K, Takagishi H, Inoue-Murayama M, Takahashi H, Yamagishi T. (2015). Polymorphism of the Oxytocin Receptor Gene Modulates Behavioral and Attitudinal Trust among Men but Not Women. *PLoS One*, 10, e0137089. |
| 14. | Yamagishi T, Li Y, Takagishi H, Matsumoto Y, Kiyonari T. (2014). In search of homo economicus. *Psychol Sci*, 25, 1699-1711. |

| Table S2 Correlation coefficients of prosocial behavior between each economic game. | | | | | | | | | | | |
| --- | --- | --- | --- | --- | --- | --- | --- | --- | --- | --- | --- |
|  |  | 1 |  | 2 |  | 3 |  | 4 |  | 5 |  |
| 1 | Dictator game | - |  |  |  |  |  |  |  |  |  |
| 2 | Prisoner's dilemma game I | .601 | ^*^ | - |  |  |  |  |  |  |  |
| 3 | Prisoner's dilemma game II | .600 | ^*^ | .486 | ^*^ | - |  |  |  |  |  |
| 4 | Public goods game | .605 | ^*^ | .499 | ^*^ | .677 | ^*^ | - |  |  |  |
| 5 | Trust game | .726 | ^*^ | .577 | ^*^ | .671 | ^*^ | .576 | ^*^ | - |  |
| * *p* <.0001 | | | | | | | | | | | |
